# Investigation of stemness and multipotency of equine adipose-derived mesenchymal stem cells (ASCs) from different fat sources in comparison with lipoma

**DOI:** 10.1186/s13287-019-1429-0

**Published:** 2019-10-22

**Authors:** Stefan Arnhold, Mohamed I. Elashry, Michele C. Klymiuk, Florian Geburek

**Affiliations:** 1 Institute of Veterinary Anatomy, Histology and Embryology, Justus-Liebig-University of Giessen, Frankfurter Str. 98, 35392 Giessen, Germany; 2 Anatomy and Embryology Department, Faculty of Veterinary Medicine, University of Mansoura, Mansoura, 35516 Egypt; 3 Clinic for Horses, University of Veterinary Medicine Hannover, Foundation, Hannover, Germany

**Keywords:** Mesenchymal stem cells, Lipoma, Cell proliferation, Cell differentiation

## Abstract

**Background:**

Adipose tissue-derived mesenchymal stem cells (ASCs) offer a promising cell source for therapeutic applications in musculoskeletal disorders. The appropriate selection of ASCs from various fat depots for cell-based therapy is challenging. The present study aims to compare stemness and multipotency of ASCs derived from retroperitoneal (RP), subcutaneous (SC), and lipoma (LP) fat to assess their usefulness for clinical application.

**Methods:**

Equine ASCs from the three fat tissue sources were isolated and characterized. The cell viability, proliferation, and self-renewal were evaluated using MTT, sulforhodamine B, and colony forming unit (CFU) assays. Stem cell relative marker CD44, CD90, and CD105 and tumor marker CA9 and osteopontin (OPN) expression were quantified using RT-qPCR. Multipotency of ASCs for adipogenic, osteogenic, and chondrogenic differentiation was examined by quantifying Oil Red O and Alizarin Red S staining, alkaline phosphatase activity (ALP), and expression of differentiation relative markers. All data were statistically analyzed using ANOVA.

**Results:**

RP fat-derived ASCs showed a higher cell proliferation rate compared to SC and LP derived cells. In contrast, ASCs from lipoma displayed a lower proliferation rate and impaired CFU capacities. The expression of CD44, CD90, and CD105 was upregulated in RP and SC derived cells but not in LP cells. RP fat-derived cells displayed a higher adipogenic potential compared to SC and LP cells. Although ASCs from all fat sources showed enhanced ALP activity following osteogenic differentiation, SC fat-derived cells revealed upregulated ALP and bone morphogenetic protein-2 expression together with a higher calcium deposition. We found an enhanced chondrogenic potency of RP and SC fat-derived cells as shown by Alcian blue staining and upregulation of aggrecan (Aggre), cartilage oligomeric matrix protein precursor (COMP), and collagen 2a1 (Col2a1) expression compared to LP. The expression of OPN and CA9 was exclusively upregulated in the ASCs of LP.

**Conclusions:**

The results provide evidence of variation in ASC performance not only between normal fat depots but also compared to LP cells which suggest a different molecular regulation controlling the cell fate. These data provided are useful when considering a source for cell replacement therapy in equine veterinary medicine.

## Introduction

Adipose tissue-derived MSCs (ASCs) are a promising cell source in regenerative medicine in terms of their therapeutic applications for musculoskeletal disorders involving bone, cartilage, and tendon lesions [1]. Currently, tissue engineering strategies including cell therapies for acute and chronic disorders by using different MSC sources are gradually becoming routine applications in clinical settings [2].

There is clear biological evidence from human and equine studies that a higher number of MSCs can be isolated from adipose tissue compared to similar amounts of bone marrow [3, 4]. In contrast to bone marrow, subcutaneous adipose tissue is easier to harvest and the sampling technique is associated with fewer risks [5]. Another detrimental factor is stemness, meaning the long-term self-renewal and multipotential differentiation of MSCs [6]; it has been shown that bone marrow MSCs exhibited signs of cell senescence at passage 7; however, ASCs can be cultivated up to passage 8 without any signs of senescence [7]. Using appropriate isolation techniques, ASCs can be efficiently purified and expanded under in vitro culture condition for further application.

Like their bone marrow-derived counterparts, ASCs from different species showed a broad spectrum of differentiation potentials [8, 9]. From the morphological point of view, ASCs have a spindle-shaped fibroblast-like appearance in culture. However, upon isolation, the number of harvested ASCs can vary between donors. Their number correlates negatively to the age of the donors, and it depends not only on their health status [10] but also on the tissue of origin [11]. Equine ASCs are usually harvested after excision or aspiration of subcutaneous fat from the paracoccygeal region in the standing sedated horse under local analgesia.

However, due to the potential therapeutic use of allogeneic MSCs, storage of autologous cells for potential future use, donor site morbidity, and other factors, alternative anatomic sites for harvesting adipose tissue should be considered in horses. In this context, emergency or elective laparotomy under general anesthesia might offer a new window to retroperitoneal and other fat sources such as lipomas. At the same time, alternative harvesting sites require an individual evaluation of the cell yield, viability, stemness, and the differentiation potential of the isolated ASCs population. These parameters are mandatory before the therapeutic application of ASCs from alternative sources can be supported. It has been reported that a large amount of subcutaneous fat can be collected via either lipectomy or liposuction in humans and horses safely and with invasive surgical approaches with limited invasiveness [12]. Based on data obtained from humans, marked differences between varying fat depots used for ASC isolation have to be considered with regard to the cell evaluation parameters mentioned above [13]. It was found that subcutaneous (SC) fat-derived cells show a higher proliferation rate in comparison to cells from the omental region in humans [14]. A similar study compared the chondrogenic effect of ASCs isolated from SC and visceral (VC) fat in a rat osteoarthritis model. Although cells from VC fat showed a higher proliferation-potential rate, cells from SC fat exhibited superior chondrogenic potential and immunosuppressive activity suggesting a usefulness of SC-ASCs for the treatment of osteoarthritis [15]. Thus, the evaluation of stem cell performance parameters including cell viability, proliferation rate, colony formation, and differentiation capacities for each alternative fat source is important before establishing an allogenic cell source for clinical purposes.

Commonly, MSC isolation and selection was based on the expression of specific relative markers, and it was reported that MSC identification relies on the expression of CD105, CD90, and CD73 and absence of CD34, CD45, CD14, and major histocompatibility class II (MHC-II) expression [16]. The differences in the surface marker expression between MSCs derived from various tissues were also previously monitored. In this context, it was found that ASCs are positive for CD34 while the expression of this marker was not existent in MSCs from bone marrow [17–19].

Lipomas (LPs) have been reported as an alternative to SC fat for ASC isolation [20]. Lipomas are benign tumors of adipose tissue and represent one of the most common soft tissue neoplasms of mesenchymal origin in humans [21, 22]. In man, lipomas can occur either as localized nodules or in the form of generalized lipomatosis. The latter is characterized by slowly growing, diffuse accumulations, or encapsulated nodules of adipose tissue formed by a heterogeneous cell population [23]. In horses, pedunculated lipomas are a common cause of intestinal strangulating obstruction encountered during emergency laparotomies [24] while the prevalence of lipomas in other anatomic sites is low. To the authors’ knowledge, ASCs from equine lipomas have not been characterized with regard to their cell viability, proliferation pattern, stemness, and other characteristics.

The current knowledge about human lipoma-derived ASCs implies some discrepancies compared to ASCs from other sources regarding their proliferation and differentiation pattern [25]. On the one hand, it has been reported in humans that lipoma-derived cells are a valuable source for tissue regeneration as they exhibit a similar proliferation and adipogenic differentiation pattern compared to ASCs [26]. In contrast to that, other reports revealed a different morphology, proliferation pattern, and other biological characteristics between ASCs and lipoma-derived cells [20].

Preperitoneal or retroperitoneal (RP) fat is another, less explored depot of white fat which is encountered and severed during routine ventral midline laparotomy in horses.

The aim of the present investigation was to investigate the differences between ASCs derived from subcutaneous (SC) and retroperitoneal (RP) adipose tissue on the one hand and lipomas (LP) on the other hand.

It was hypothesized that the ASCs harvested from equine lipomas have a higher cell proliferation and differentiation capacity predestinating them as an alternative for tissue regeneration compared to MSCs from subcutaneous and retroperitoneal fat.

Thus, the performance of ASCs harvested from SC, RP, and LP fat was examined (1) in terms of cell viability, proliferation pattern, and stemness as indicated by colony forming unit (CFU) assay and surface markers’ expression. (2) The multipotency of ASCs into the adipogenic, osteogenic, and chondrogenic differentiation lineages was verified by quantifying Oil Red O and Alizarin Red S staining, measuring alkaline phosphatase (ALP) activity and histological means using Alcian blue staining.

The current study provides evidence of a higher proliferation rate and more adipogenic capacity of RP fat-derived ASCs compared to cells from SC and LP. LP derived cells were found to have an impaired multipotency towards various differentiation fate. An enhanced ALP activity was detected in all cell sources; however, SC fat-derived cells revealed a higher osteogenic capacity as indicated by ARS quantification and upregulation of alkaline phosphatase (ALP) and bone morphogenetic protein-2 (BMP2) expression up to day 14. The higher chondrogenic potential of SC and RP derived cells as shown by Alcian blue staining and upregulated aggrecan (Aggre), cartilage oligomeric matrix protein precursor (COMP), and collagen 2a1 (Col2a1) expression emphasized their usefulness for cartilage repair. In addition, the upregulation of the tumor cell marker CA9 and osteopontin (OPN) expression exclusively in the LP derived cells underlines their neoplastic background.

## Materials and methods

### Isolation and cultivation of equine ASCs

Subcutaneous (SC) adipose tissue was collected by excision from the paracoccygeal region, i.e., above the *M. gluteus superficialis* as previously described [27], and from the retroperitoneal (RP) space in the region of the post umbilical ventral midline. Study horses included mares and geldings of different breeds and had mean age of 4.75 ± 1.71 years. While the subcutaneous fat samples (*n* = 8) were obtained from horses euthanized due to reasons not related to this study, samples from retroperitoneal fat (*n* = 8) and mesenteric lipomas (LP; *n* = 8) were obtained from horses undergoing abdominal surgery at the Clinic for Horses, Department of Surgery, at the Faculty of Veterinary Medicine, Justus-Liebig-University of Giessen. All the standard procedures were approved by the local authorities (RP Giessen) regarding animal care and use.

After harvesting, adipose tissue from the different collection sites was diced into small pieces and washed in an equal volume of phosphate-buffered saline (PBS, Gibco, Germany) supplemented with 1% penicillin/streptomycin (P/S, AppliChem). For cell isolation, the adipose tissue was cut using a sterile scalpel-blade, then undergone enzymatic digestion using 0.1% collagenase type I (Biochrom AG, Germany) dissolved in 1% bovine serum albumin (PAA, Germany) in PBS at 37 °C with mild shaking for 30 min. The digested fat homogenate was filtered through a 70-μm nylon cell strainer mesh, then was centrifuged at 260*g* for 5 min. The cell pellet was washed in PBS, centrifuged at 300*g* for 5 min, and was suspended in fresh 10% fetal calf serum (FCS, Capricorn/DMEM, Gibco Life technologies). After cell counting using a hemocytometer, cells from all sampling sites were cultivated in a culture dish at a density of 2.5 × 10^5^ cells per cm^2^. After 24 h, the cultures flasks were washed with PBS to remove the non-adherent cells, and the medium was replaced three times per week. Up on 80% confluency, the cells were detached from the culture dish using TrypLE Express Enzyme (Thermo Fisher Scientific), were washed in fresh medium, were counted, and were plated according to the experimental setup.

### Cell count

To get a direct information about the proliferative capacity, cells of passage (P2 to P5) were plated at a density of 5 × 10^5^ cells/well. After the cultivation period, cells were detached and were counted using a hemocytometer.

### Fluorescence-activated cell sorting (FACS) analysis

To sort out the ASCs harvested from various adipose tissue based on the positivity for the stem cell-specific markers, FACS analysis was carried out. Briefly, 2 × 10^6^ cell suspension per mL in fresh medium was prepared. A volume of 100 μL of cell suspension per well was transferred into a 96-round-bottomed-well-culture plate. The plate was centrifuged at 400*g* for 3 min at room temperature. The supernatant was carefully discarded without disturbing the cell pellet. The pellets were resuspended in 100 μL of washing buffer containing 99% PBS+1% bovine serum albumen (BSA) supplemented with 0.01% NaN3 and 0.5% goat serum and 10% horse serum, then were centrifuged at 400*g* for 3 min at room temperature. The pellets were incubated with 50 μL of the primary antibodies for 20 min at room temperature, then were centrifuged at 400*g* for 3 min. After the supernatant was discarded, the cells were washed twice using the washing buffer for 3 min and were centrifuged at 400*g* for 3 min. The cells were incubated with 50 μL of the secondary antibody for 20 min in dark. After two times washing, the pellets were resuspended in PBS for FACS analysis (Accuri C6®, BD Bioscience, Heidelberg, Germany) equipped with Accuri C6 software (BD Bisoscience, Heidelberg, Germany).

### MTT cell viability assay

MTT assay was performed after 48 h to investigate the cell viability of ASCs from the different adipose tissue sources. ASCs were seeded at a density of 1 × 10^5^ cells/well in 24-well-culture plates in triplicates. As vital cells are capable of reducing the yellow MTT (3-(4, 5-dimetylthiazol-2-yl)- 2, 5-diphenyltetrazolium bromide) to the purple formazan, the cells were incubated with the MTT solution (5 mg/mL) dissolved in PBS added to fresh medium at 37 °C and 5% CO_2_. After 3–4 h of incubation, the medium was removed and a volume of 200 μL per well of dimethyl sulfoxide (DMSO, Roth, Germany) was added for 10 min. Optical density of the formazan crystals was measured at 570 nm to determine the relative number of cells using a TECAN Sunrise plate reader (TECAN).

### Sulforhodamine B (SRB) protein assay

In order to semi-quantify the cellular proteins contents as an indicator for cell number, the SRB, a colorimetric assay, was carried out as previously described [28]. Briefly after 48 h of cultivation, cells from all sampling sites were fixed in 4% paraformaldehyde (PFA, Roth, Germany) and were incubated with 2 mL per well of 4% (w/v) sulforhodamine B dissolved in 1% acetic acid solution at room temperature for 10 min. SRB was removed, and the plates were rinsed 5 times for 5 min with 1% (v/v) acetic acid to remove the unbound staining. A volume of 2 mL per well of 10 mM unbuffered Tris-based solution (pH 10. 13) was added, and the plates were left on a plate shaker to dissolve the bound protein stain for approximately 30 min. A volume of 100 μL aliquots from each experimental condition were transferred into 96-well plate. The absorbance was measured at 565 nm using TECAN Sunrise plate reader (Tecan, Germany).

### Colony forming unit (CFU) assay

The self-renewing potency of cells isolated from lipomas as well as from subcutaneous and retroperitoneal fat was studied using a CFU assay which was carried out according to an established method [29]. For each adipose sample, cells from the third passage were plated at two cell densities: 50 and 100 cell per T25 cell culture flask (Thermo fisher). Cells were incubated in DMEM growth medium with 10% FCS with medium change twice per week. After 8-day incubation period, cells were rinsed in PBS, fixed in 4% PFA for 20 min, and were stained with 1% crystal violet (Sigma-Aldrich) dissolved in 100% methanol (Roth, Germany) for 10 min. After then, the cells were washed in PBS three times and were air-dried overnight. For all experimental groups, the colonies of more than 50 cells were counted using the inverted light microscope.

### Real-time quantitative polymerase chain reaction (RT-qPCR)

Total RNA was extracted from a minimum of 5 × 10^5^ cells using an innuprep RNA-mini kit according to the manufacturer’s protocol (Jena Analytik, Germany). Briefly, 1 μg RNA from all experimental groups were treated with a recombinant DNAse I (Roche) and RNase inhibitor (Thermo fisher scientific) subsequently were reverse transcribed (RT) in one step using a multiscribe reverse transcriptase (Applied Biosystems), RNAase inhibitor and reverse transcription mix according to the manufacturer’s protocol (Applied Biosystems). Minus RT samples without reverse transcriptase for each specimen were included as experimental negative controls. To examine the stem cell surface markers as well as the relative differentiation gene expression, PCR was conducted according to the manufacturer’s protocol. All primers were random hexamers purchased from Microsynth (Germany). Primers for the following genes were used: CD 44, CD 90, CD 105, CD 45, OP, and CA9 (Table 1). To test the primers efficiency and PCR cyclic conditions, a qualitative PCR was carried out as follows; 95 °C for 10 min, following by 39 cycles of 95 °C for 1 min, 60 °C for 1 min, 72 °C for 90 s and finally 72 °C for 10 min. PCR products were verified in 2% agarose gel electrophoresis labeled with SYBER Green (Sigma, Germany) and visualized by using UVI doc software (Biometra, Germany). In order to evaluate ASC multipotency, quantitative PCR for ALP and BMP-2, fatty acid binding protein-4 (FABP4), peroxisome proliferator-activated receptor gamma (PPARγ) and leptin (LEP), and Aggre, COMP, and Col2a1 was carried out up to day 14 post osteogenic, adipogenic, and chondrogenic differentiation. Briefly, 2 μL of transcribed cDNA from RP, SC, and LP differentiation-induced cells was mixed with 10 μL SYBER green qPCR-master mix (Promega) with 10 pM/μL forward and reverse primers (Table 1) in triplicates. The reaction was performed for 35 cycles of 5 min at 95 °C, 30 s at 94 °C, 30 s at 60 °C, and 30 s at 72 °C using Bio-Rad real time PCR detection system (Bio-Rad, Munich, Germany). Gene expression was normalized to the house keeping gene glyceraldehyde 3-phosphate dehydrogenase (GAPDH) and 18S. In parallel, a negative control without cDNA was used to assess the PCR efficiency. Relative gene expression was normalized to *GAPDH* and *18S* references gene using the 2^−∆∆CT^ method as well as previously described [30].

**Table 1:** List of the primers used for PCR

Primer	Forward	Reverse	Product size
CD105	CCGCCGCACTGTGGTACATCTAC	TGTGGTTGGTGCTACTGCTCTCTG	108
CD90	CCCGTGGGCAGAAGGTGAC	TCAGGCTGAACTCATACTGGATGG	112
CD44	CAGAACCAACCCTGAAGACATC	GAAGGTGGGTGTGGAAGATG	116
CD45	GTTACGTTGACATCCTTCCTTA	GCAATGTATTTCCTGGGTTCTTTG	127
ALP	GACTGGTACTCGGACAACGAG	GTTCTTGGGGAACATGTACTTC	137
BMP2	CAACACGGTGCGCAGCTTC	CGCCGGGTTGTTTTTCCAC	74
Aggre	CCTCATCGGAAACCTATGATGTCT	GTTGTGGGACCCACAGAACTC	122
COMP	GCAGGACTCAGACAGCGATGG	GGCACCAGGCGGCAGTTG	96
Col2a1	CTGCCCATCATTGACATTGCAC	GTCCACACCAAATTCCTGCTC	63
FABP4	ATCAGTGTAAACGGGGATGTG	GACTTTTCTGTCATCCGCAGTA	117
LEP	CGAAAAGTCCAGGATGACAC	AACCAGTGACCCTCTGTTTG	106
PPARγ	GTCTCATAACGCCATCAGGTTTG	GCCCTCGCCTTCGCTTTG	180
OP	TGATCCTACTGATGACCCTGAC	ATACGTGTCTCTTGTGGGCAC	136
CA9	TGCAACTGCTGCTGTTACTG	TCTTCCCCAGATGAATCTCC	103
GAPDH	CCAGCAAGAGAAGGAGAAAGG	AACTGTGGAGGTCAGGAGAC	93
18S	ATGCGGCGGCGTTATTCC	GCTATCAATCTGTCAATCCTGT	204

### Immunofluorescence of stem cell-specific markers

**Fig. 1:**
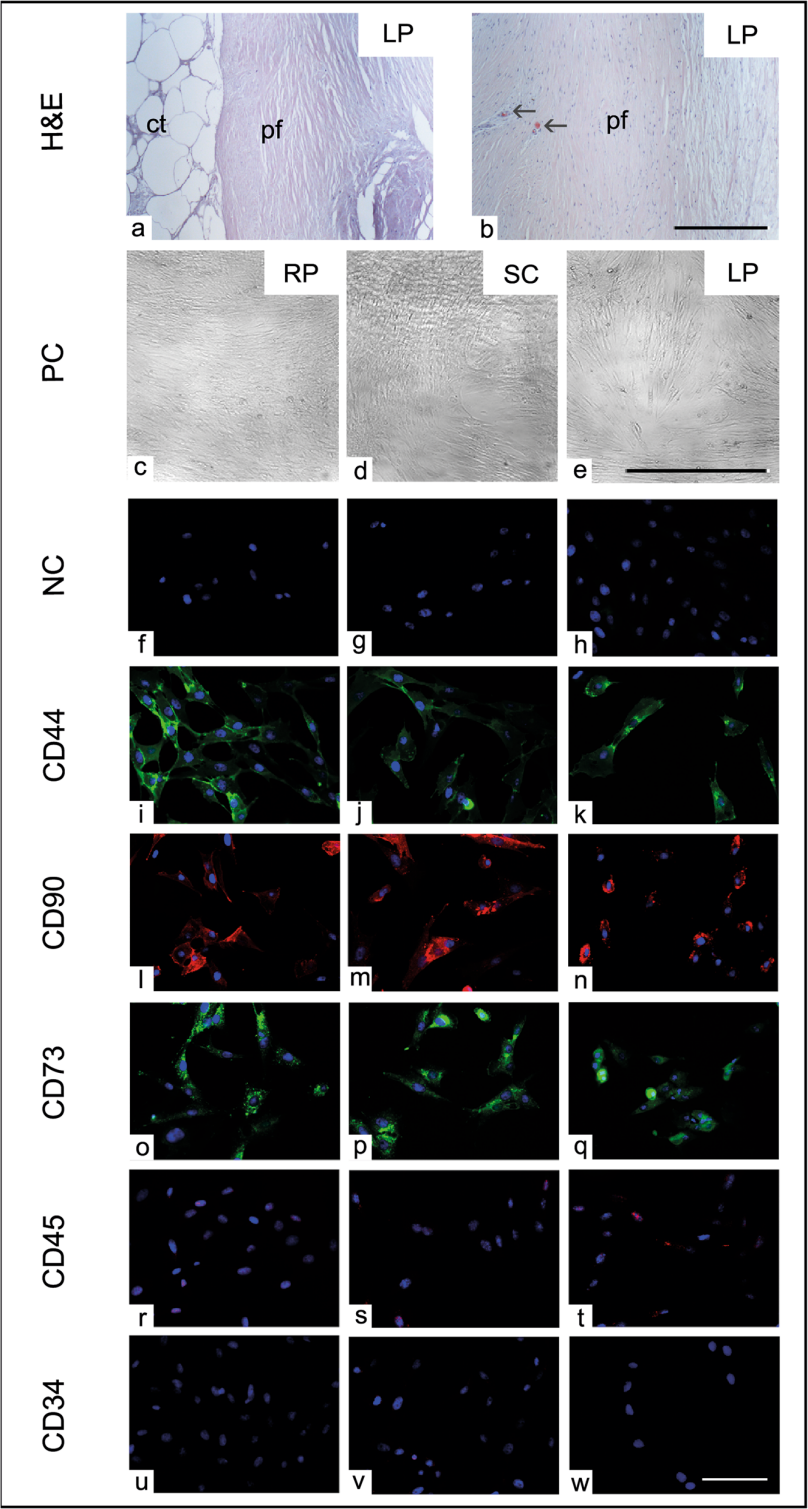
Morphology of lipoma-derived cells. **a**, **b** Histological 5-μm paraffin section of lipoma (LP) stained with hematoxylin and eosin (H&E). **a** The central part (ct) of LP shows the typical morphology of the adipose tissue while the peripheral capsule was formed of dense connective tissue layer. **b** Lipoma capsule (pf) shows longitudinally oriented collagen fibers with numerous capillaries (black arrow). **c**–**e** Phase contrast (PC) images of ASCs harvested from retroperitoneal (**c**, RP), subcutaneous (**d**, SC), and lipoma (**e**, LP) adipose tissue exhibit the typical spindle-shaped fibroblast-like morphology of stem cells under culture condition. (**f**–**w**) Immunofluorescence images show CD44 (green, **i**–**k**), CD90 (red, **l**–**n**), and CD73 (green, **o**–**q**) positive ASCs of RP, SC, and LP fat, respectively. **r**–**w** Immunofluorescence images show CD45 (**r**–**t**) and CD34 (**u**–**w**) negative ASCs of RP, SC, and LP fat, respectively. Cells from all experimental groups with no added primary antibodies were processed in parallel and were served as negative controls (NC, **f**–**h**). Scale bar in **a**, **b** = 0.5 mm, **c**–**e** = 100 μm, and **f**–**w** = 20 μm

To examine the distribution of stem cell markers, immunofluorescence for ASCs harvested from RP, SC, and LP fat was performed. Briefly, 1 × 10^4^ cells per well were cultivated on a glass cover slips in 24-well culture plate using growth medium. After 24 h, the cells were washed twice in PBS for 5 min and were fixed in 4% PFA for 10 min at room temperature. The cells were permeabilized with 0.02% Tween-20 in PBS for 10 min, were washed twice in PBS, then were blocked in 5% FCS for 30 min. The cells were incubated with mouse anti CD90 1:50, CD44 1:20, CD45 1:50, CD43 1:50, MHC type II 1:50, and goat polyclonal anti CD73 1:50 primary antibodies overnight at 4 °C. The immunoreaction was visualized by incubating the cells with goat anti-mouse fluorescent isothiocyanate (FITC, 1:100, Dianova), anti-mouse IgG Cy3 (1:100, Dianova), and donkey antigoat alexaflour 488 (1:200, ThermoFisher) secondary antibodies for 1 h in the dark. The cell nucleus was detected using 4′,6-diamidin-2-phenylindol (DAPI, ThermoFisher). The cover slips with adherent cells were carefully mounted on a glass slides, and the cells were photographed using Axio-imager fluorescent microscope equipped with a digital camera (Zeiss, Germany). Cells from all experimental groups were processed in parallel with no added primary antibodies and were served as negative controls (Fig. 1f–h).

### Induction of adipogenic differentiation

To investigate the ASCs’ adipogenic differentiation potential, the cells of different adipose tissue samples were grown on a glass coverslip and were incubated with adipogenic differentiation medium (ADM) consisting of high glucose DMEM, 5% FCS, 1% P/S, 0.1 μM dexamethasone (Sigma, Aldrich), 0.5 mM IBMX (Sigma, Aldrich), 5 μg/ml insulin-transferrin-selenium (ITS), and 5 μM rosiglitazone (Sigma) for 10 days. In parallel, cells incubated in the basal medium (BM) including low glucose DMEM, 5% FCS, and 1% P/S were used as a negative control. After the induction time, the cell population was fixed in 4% PFA, washed in PBS, and was incubated with Oil Red O (ORO, Sigma-Aldrich) staining diluted in distilled water for detection of the intracellular lipid droplets. Cell nuclei were visualized using hematoxylin (Merck) for 10 min. ORO stained cells were mounted on a glass slides using Kaiser’s glycerol gelatin (Merck) and were examined and photographed by the inverted light microscope equipped with a digital camera and the LAS V4.4 software (Leica, Germany). To semi quantify the adipogenic potential of ASCs, ORO bound staining were extracted from the cells using 100% isopropanol for 30 min. For each experimental group, the absorbance was measured in 96-well microplate at 492 nm in triplicates using (Tecan, Germany).

### Induction of osteogenic differentiation

To evaluate the osteogenic potential of ASCs isolated from subcutaneous, retroperitoneal and lipoma fat, osteogenic differentiation was carried out as described previously by [27]. Briefly, the cells were seeded in 24-well plates (VWR, Germany) at a density of 1 × 10^5^ cells per well. After 80% confluency was achieved, osteogenic differentiation induction using was performed using standard osteogenic differentiation medium (ODM) composed of 100 nm dexamethasone (Sigma-Aldrich, Germany), 0.05 mM ascorbic acid (Sigma-Aldrich, Germany), 10 mM β-glycerol phosphate (Sigma-Aldrich, Germany) in DMEM supplemented with 5% FCS and 1% P/S. No other stimulating factors relevant to the osteogenic differentiation were provided. For each experimental setup, cells grown in triplicates in a basal medium (BM), composed of DMEM with 5% FCS and 1% P/S, were served as a negative control. The cells were incubated for 7, 14, and 21 days in a humidified culture condition at 37 °C and 5% CO_2_. The cells were provided with a fresh medium twice a week.

### Induction of chondrogenic differentiation

To examine the chondrogenic potential of ASCs harvested from RP, SC, and LP fat, chondrogenic induction was performed using 1 g/mL DMEM medium including 0.1 μM dexamethasone, 10 μg/mL insulin-transferrin selenium (ITS), 100 μg/mL sodium pyruvate, 50 μg/mL ascorbic acid, 40 μg/mL prolin, and 10 ng/mL transforming growth factor-β (TGF-β). Briefly, ASCs from all experimental groups were counted and 1 × 10^5^ cell/mL in DMEM with 10% FCS and 1% P/S were cultivated in 96-well plates in triplicates. The cells were centrifuged at 100*g* for 5 min. After 48-h incubation, the cell pellets were incubated in fresh chondrogenic medium with changing twice a week up to day 21 post induction. The cell pellets were transferred into PCR tubes and were washed twice with distilled H_2_O and were fixed in 4% PFA for 1 h at days 7, 14, and 21. The cell pellets were embedded in paraffin, sectioned at 5 μm, and processed for histological examination using 1% Alcian blue (Sigma-Aldrich) staining for 30 min. The sections were photographed at × 5 objective using an ordinary light microscope equipped with a digital camera and the LAS V4.4 software (Leica, Germany).

### Semi-quantification of alkaline phosphatase (ALP) activity

The cells from different fat sources undergoing osteogenic differentiation up to day 14 were examined. Briefly, the media were discarded and the cells were incubated with 500 μL of 1% Triton™ X-100 in 10 mM Tris (pH 7.4) at 4 °C for 1 h. The cells were detached using a cell scraper, and the lysates were centrifuged at 28,400*g* for 2 min and kept at 4 °C. P-Nitrophenylphosphate (2 mg/mL) was dissolved into buffer solution containing 1 M Tris and 5 mM MgCl_2_ (pH 9.0). A volume of 150 μL of p-nitrophenylphosphate was mixed with 50 μL of cell lysate and was loaded in 96-well microplates in triplicates. The standard curve of p-nitrophenol solution was prepared in triplicates. The activity of ALP metabolizes the p-nitrophenylphosphate substrate into p-nitrophenol (PNP) as previously described [31]. The activity of ALP was measured by the change in the color of PNP from colorless to yellow. The absorbance of PNP was measured at 405 nm using a microplate reader.

### Detection of osteogenic differentiation by Alizarin Red stain (ARS)

For the detection of osteogenic differentiation, a phase-contrast microscopic examination was carried out to track the morphological changes indicative for osteogenic commitment. In addition, ARS for the detection of calcium ion deposition as an indicator for osteogenesis was applied. Briefly, cells were grown in monolayers with a cell density of 1 × 10^5^ cells per cm^2^ in 24-well plates for 7, 14, and 21 days post differentiation induction. The cells were fixed in 4% PFA in PBS for 10 min, washed twice with PBS, and followed by three times with distilled water. The cells were incubated with 2% Alizarin Red staining solution (Roth, Germany) for 10 min at room temperature. The staining solution was removed, and the cells were washed 3–4 times with distilled water to remove unbound staining. The cellular aggregation revealing osteogenic commitment referred as osteogenic nodules was stained in orange-red with ARS due to calcium deposition. The cells were photographed using an ordinary inverted light microscope equipped with a digital camera and the LAS V4.4 software (Leica, Germany).

### Analysis for Alizarin Red S (ARS) staining

After ARS staining, the cells were washed in distilled water and were incubated with 2 mL of 10% Cetyl Pyridinium Chloride (CPC, Roth Germany) with shaking for almost 1 h. For each experimental group, 200 μL was transferred into a 96-well plate. The absorbance was measured at 562 nm in triplicates using a microplate reader (Tecan, Germany).

### Statistical analysis

To evaluate the data collected from MTT, SRB, CFU assays, and surface markers expression of ASCs from different fat sources, one-way ANOVA was performed. To analyze ALP activity as well as expression of osteogenic (ALP and BMP2) and chondrogenic (Aggre, COMP and Col2a1) genes between the different experimental groups (RP vs. SC vs. LP) at day 7 and day 14 post induction, a two-way ANOVA was carried out. To assess the expression of FABP4, PPARγ, and LEP in all experimental groups at day 8 post adipogenic differentiation, a one-way ANOVA was carried out. Multicomparison and variable interaction were performed using Tukey’s and Sidak’s post hoc tests. Data from triplicates were presented as the mean ± SEM. The data values with *p* ≤ 0.05 were considered to be significant. All the data analysis and presentation were carried out by using the Graph Pad Prism 7.0 statistical software (La Jolla, CA, USA).

## Results

### Morphology of lipoma-derived cells

The histological observation of the LP revealed dense compact mass with an outer fibrous capsule and inner adipose matrix (Fig. 1a). The outer connective tissue layer showed a dense connective tissue with collagen fibers rich in blood vessels (Fig. 1b). Harvested ASCs (passage 0) from different fat sources showed morphological differences. As expected, the cells from both fat provenances (RP and SC) exhibited the typical stem cell appearance with spindle-shaped fibroblast-like morphology (Fig. 1c, d); also, the cells isolated from lipoma shared the same morphological characteristics (Fig. 1e). After plating of 0.5 million cells per well in 24-well plate, the cells were counted. The proliferation pattern of cells from both fat provenances showed a gradual increase from passage 1 up to passage 10. However, it became obvious that numbers of lipoma-derived cells (LP) were continuously decreasing and cell growth was only maintained up to passages 5–6. In the last passage, cells from lipoma already showed signs of degeneration (Fig. 1). To examine and compare stem cell markers’ distribution in the ASCs harvested from all experimental groups, immunofluorescence of CD44, CD90, CD73, major histocompatibility complex class II (MHCII), CD45, and CD43 was carried out. As expected, the majority of cells demonstrated positive immunoreaction for CD44, CD90, CD73, and MHCII proteins (Fig. 1i–q). Additionally, ASCs showed a negative immunoreaction against CD45 and CD34 harvested from RP, SC, and LP fat (Fig. 1r–w).

### Evaluation of the cell viability and cell proliferation

**Fig. 2:**
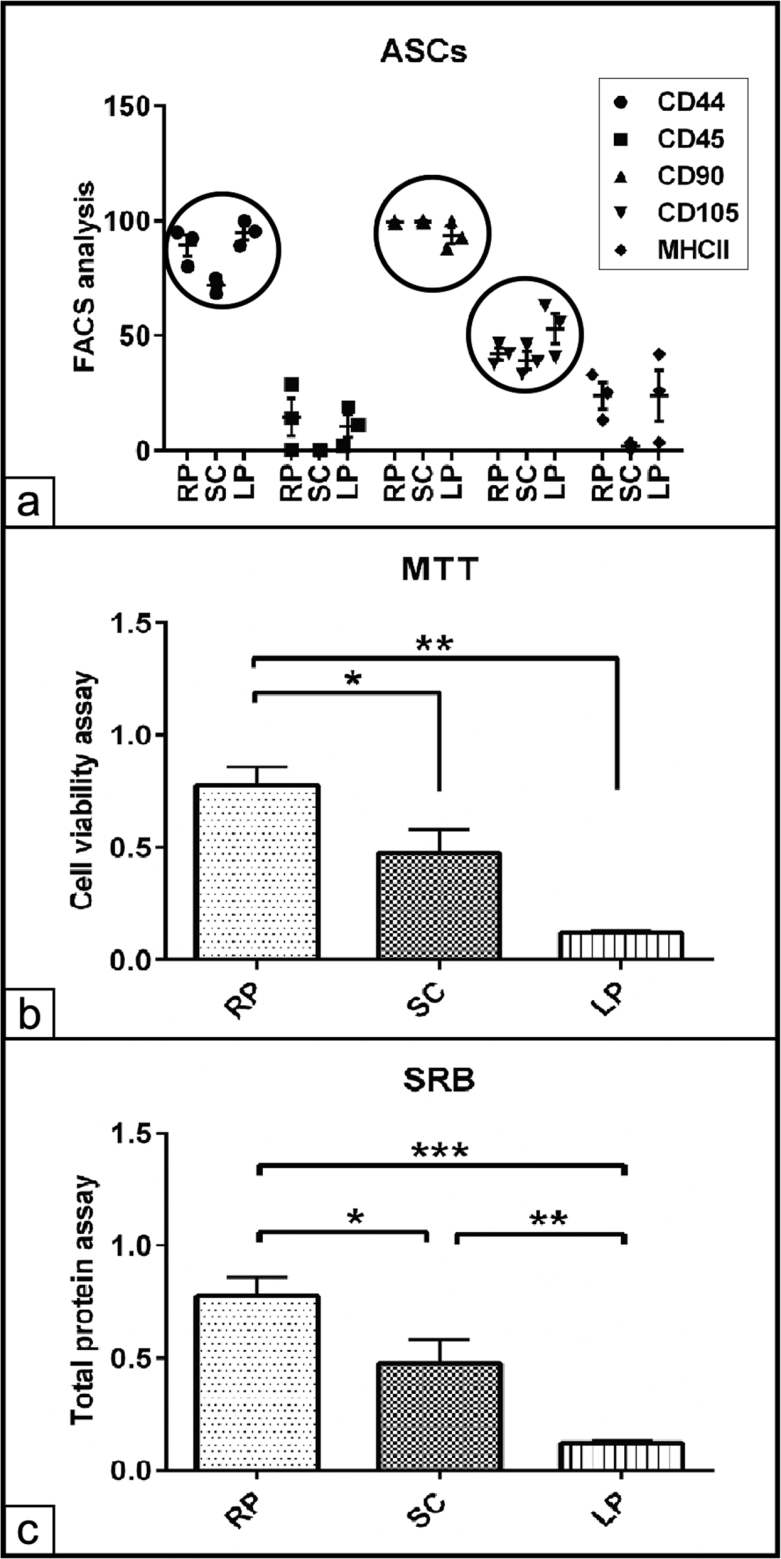
Evaluation of the cell viability and cell proliferation. **a** FACS analysis of ASCs harvested from retroperitoneal (RP), subcutaneous (SC), and lipoma (LP) adipose tissue. A volume of 100 μL of cell suspension (2 × 10^6^ per mL) per well was transferred into a 96-well-culture plate. The plate was centrifuged and the supernatant was discarded. The pellets were preblocked in 1% BSA in PBS supplemented with 0.01% NaN3 and 0.5% goat serum and 10% horse serum. The pellets were incubated with 50 μL of CD44, CD90, CD105, CD45, and MHCII primary antibodies. FACS analysis shows the percentage of CD44, CD90, and CD105 immunopositive ASCs. The selected cell populations show weak immunoreaction against CD45 and MHCII. **b** Evaluation of cell viability using MTT assay, the absorbance was measured at 570 nm. The data analysis revealed significant increases cell viability of RP fat-derived ASCs compared to both SC and LP derived cells. **c** Quantification of total protein contents of RP, SC, and LP derived ASCs cultivated under growth condition for 48 h. Sulforhodamine B assay (SRB) measures the protein contents indicative for cell number. The analysis shows higher protein contents of the RP derived cells compared to SC and LP derived cells. The SC derived cells exhibit more cells compared to LP derived cells. All data presented as mean ± SEM. *p < 0.05, **p < 0.01, and ***p < 0. 001

In order to assess and select the efficient cell populations from various fat tissues, FACS assay for RP, SC, and LP derived ASCs was performed. The analysis revealed a high immunoreactivity to the stem cell markers CD90 (99± 0.0 %, 99.6± 0.0 % and 93.5± 0.04 %) and CD44 (89± 0.05 %, 72± 0.02% and 95± 0.03% for ASCs harvested from RP, SC and LP respectively. A moderate immunoaffinity of 42 ± 0.03%, 39 ± 0.04%, and 53 ± 0.07% was observed against CD105. In contrast, ASCs demonstrated a weak immunoreaction against CD45 of 14 ± 0.08%, 0.00%, and 10.7 ± 0.05% for RP, SC, and LP, respectively. Furthermore, LP and RP derived ASCs demonstrated significant increases in CD44^+^ cells (*p* < 0.01 and *p* < 0.05) compared to those cells of SC fat (Fig. 2a). To get an overview of the cell viability and the proliferative potential of ASCs derived from different fat sources, MTT and SRB assays were carried out on the previously FACS sorted cells. The data analysis showed significant increases in the viability of ASCs isolated from RP and SC (*p* < 0.01 and *p* < 0.05) compared to those cells of LP fat. Moreover, no significant differences in the cell viability could be detected between RP and SC ASCs (Fig. 2b). On the other hand, quantification of the total protein contents indicative for cell number revealed highly significant increase in the cell number of RP derived ASCs compared to both SC (*p* < 0.05) and LP (*p* < 0.001) isolated cells. Furthermore, ASCs of SC fat displayed a higher protein contents (*p* < 0.01) compared to those cells of LP fat (Fig. 2c).

### Evaluation of stemness of ASCs from various fat sources

**Fig. 3:**
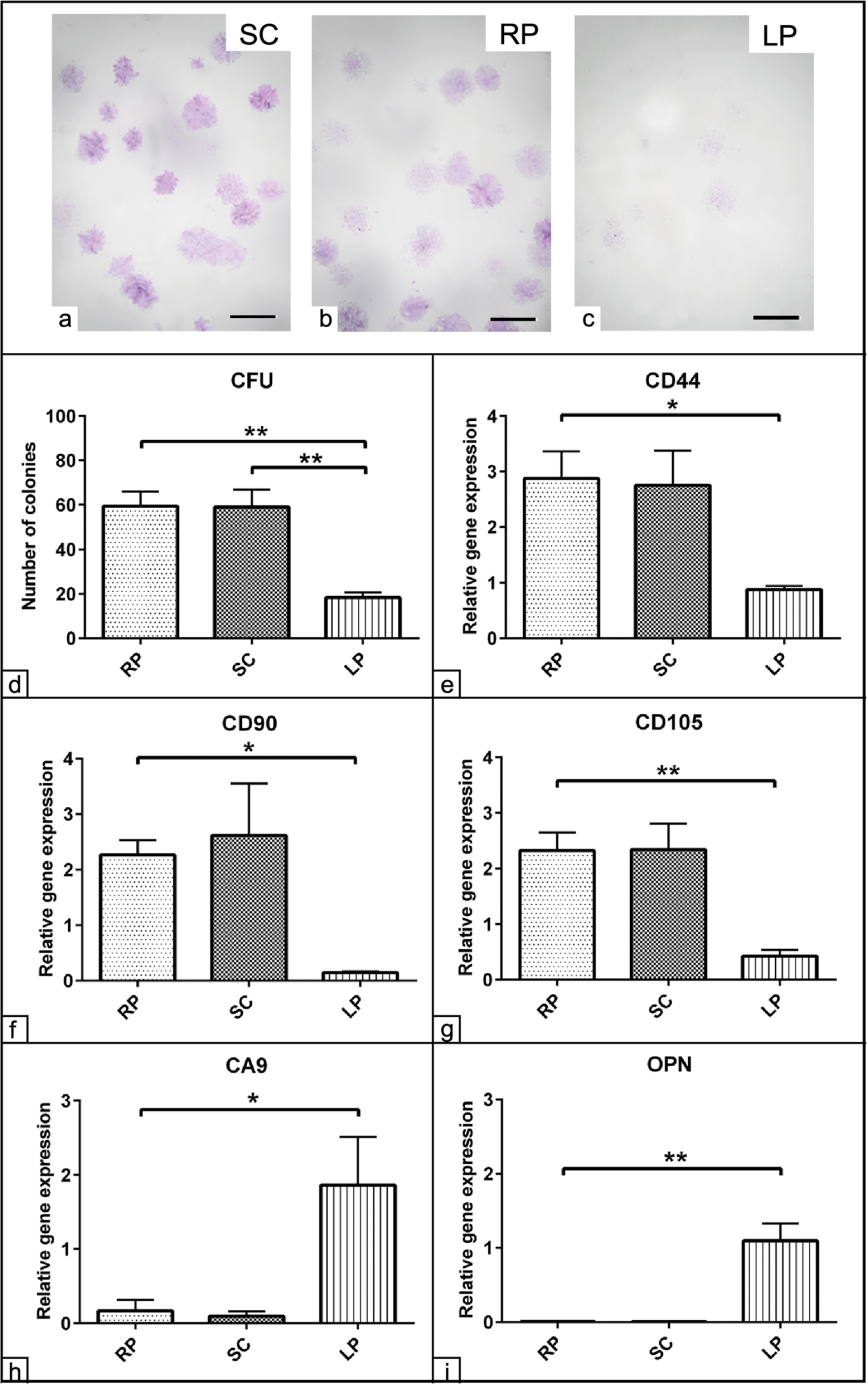
Evaluation of stemness of ASCs from various fat sources. CFU assay of subcutaneous (SC), retroperitoneal (RP), and lipoma (LP) adipose-tissue derived ASCs cultivated as 100 cells per 25 cm^2^ culture flasks for 8 days in growth medium composed of 10% FCS in DMEM and 1% P/S. **a**–**c** Representative microscopic images of SC, RP, and LP derived colonies stained with 1% crystal violet (blue) show colony formation in all experimental groups. **d** The number of 1% crystal violet stained colonies were counted. The analysis shows a marked CFU capacity of SC and RP fat-derived cells compared to those cells of LP. **e**–**i** Evaluation of mesenchymal stem relative markers expression. Subcutaneous (SC), retroperitoneal (RP), and lipoma (LP) adipose tissue-derived ASCs were cultivated under growth conditions for 48 h. A volume of 1 μg RNA per experimental group was transcribed into cDNA using a reverse transcription kit. The expression of CD44 (**e**), CD90 (**f**), CD105 (**g**), CA9 (**h**), and osteopontin (OPN, **i**) was quantified using RT-qPCR. The analysis reveals upregulation of the stem cell markers CD44, CD90, and CD105 in RP and SC derived cells compared to LP cells (**e**–**g**). Upregulation of the CA9 and OPN expression could be shown only in LP derived cells (**h**, **i**). The analysis was performed in triplicates for all experimental groups. GAPDH and 18S house-keeping genes were used as endogenous references. The data presented as mean ± SEM. *p < 0.05 and **p < 0.01. Scale bar = 5 mm

The colony forming unit (CFU) evaluation is a valid assay to measure mesenchymal stem cells’ stemness. Cells of all three fat sources were plated at 50, 100, and 200 cell per T25 culture flask for up to 8 days. While the number of colonies of cells derived from the RP and SC fat was comparable, there was a much lower number of colonies observable in cells derived from LP. Furthermore, the size of colonies seemed to be slightly bigger in cells from the RP compared to SC fat. Interestingly, colonies of LP derived cells were more divergent with a weaker staining compared to the colony forming pattern as shown in RP and SC fat (Fig. 3a–c). To quantify these morphological observations, 100 cells per flask from each experimental condition were plated, and the colonies of ≥ 50 cells were quantified. Significant increases in the average number of colonies were detected with the cells of RP and SC fat (*p* < 0.01) compared to those of the LP fat. The cells of the LP revealed almost 70% reduction in the number of colonies compared to the other fat sources (Fig. 3d). To confirm our data, the expression of stem cell-specific markers named CD44, CD90, and CD105 of non-induced ASCs from all experimental groups were quantified using RT-qPCR. The analysis showed significant upregulation of CD44 (*p* < 0.05), CD90 (*p* < 0.05), and CD105 (*p* < 0.01) expression in the ASCs of RP and SC fat compared to those cells from LP fat. Furthermore, there were no real detectable differences regarding transcript expression when cells derived from RP and SC adipose tissue were compared. In addition, the expression of these surface markers was significantly lower in the LP derived cells (Fig. 3e–g). In contrast, the expression of the marker for hematopoietic precursor cells CD45 was not detectable in the cells from all sampling sites excluding the possibility of contamination from other cell populations in terms of hematopoietic precursor cells.

The previous observation demonstrated a marked distinction between the ASCs from RP and SC on the one hand and cells from LP on the other hand. To validate these data, we have examined the expression of the tumor marker CA9 expression. The analysis revealed a strong expression of the tumor marker CA9 in LP derived cells only (*p* < 0.05) compared to those cells from RP and SC fat (Fig. 3h). Unexpectedly, late osteogenic differentiation marker, osteopontin (OPN), showed upregulation particularly in the LP derived cells (*p* < 0.01) compared to RP and SC fat isolated cells (Fig. 3i).

### Detection of ASC multipotency

**Fig. 4:**
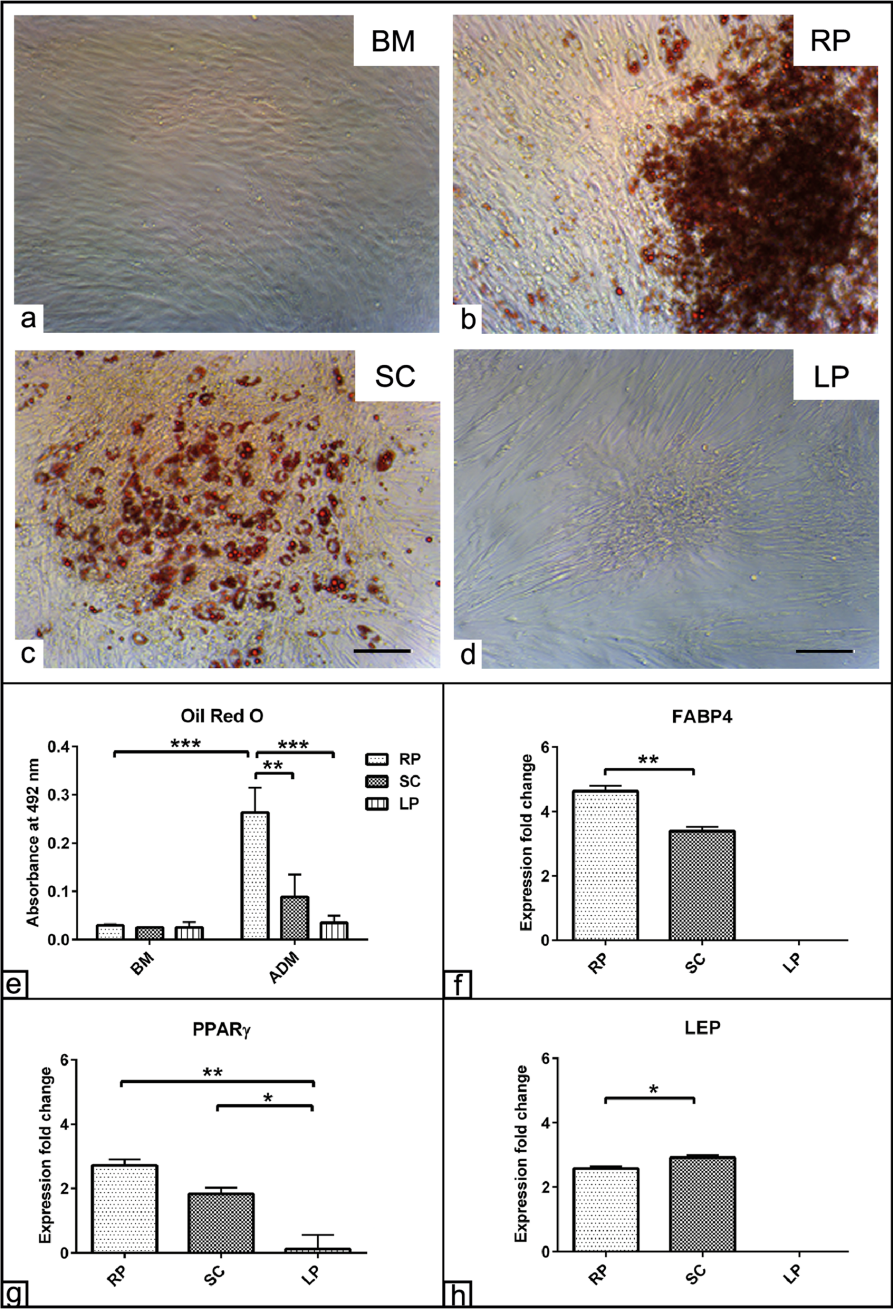
Adipogenic differentiation of ASCs. ASCs harvested from retroperitoneal (RP), subcutaneous (SC), and lipoma (LP) fat sources were cultivated in the presence of basal medium (BM). **a**–**d** Photomicrographs of RP, SC, and LP derived ASCs stained with ORO staining show fat vacuole formation (red) following adipogenic differentiation induction. The cells of LP exhibit an impaired adipogenic differentiation capacity. **e** Semi-quantification of the ORO staining RP, SC, and LP derived ASCs was performed at 492-nm absorbance. RP fat-induced cells show a higher ORO values compared to both SC and LP induced cells as well as those cells cultivated in BM. **f**–**h** Expression fold change of FABP4 (**f**), PPARγ (**g**), and LEP (**h**) at day 10 post adipogenic differentiation induction show upregulation of adipogenic markers in RP and SC derived cells compared to LP cells. The expression was normalized to non-induced cells in BM using 2^−∆∆cq^ method [30]. The data presented as mean ± SEM. *p < 0.05, **p < 0.01, and ***p < 0.001. Scale bar = 100 μm

The multipotency of stem cells from the three different adipose tissue sources was investigated by inducing the cells to differentiate into the three major mesenchymal lineages such as the adipogenic, osteogenic, and chondrogenic differentiation fate. Adipogenic differentiation was evaluated using ORO staining for tracking fat vacuoles formation. Already after 10 days post adipogenic induction, ASCs isolated from RP and SC displayed marked increases in the fat vacuole formation compared to those cells cultivated either in BM or from LP fat (Fig. 4a–d). To measure the adipogenic capacity for each fat source-derived cell, ORO staining was solubilized and the absorbance was measured. The analysis showed an intensive adipogenic potential of RP derived cells as indicated by the values of ORO quantification compared to those cells cultivated in BM (*p* < 0.001) as well as by comparing with those cells from SC (*p* < 0.01) and LP fat (*p* < 0.001). Although there was a marked increase in the ORO value in the cells of SC fat, it was not statistically significant. In contrast, the values of ORO staining of LP derived cells were too low to be quantified even after 10 days of adipogenic induction (Fig. 4e). The analysis also detected significant interaction (*p* < 0.01) between the effect of ADM and the cell source. These data suggest that the effect of an adipogenic induction was dependent on the cell origin and moreover that ASCs derived from RP demonstrated a superior potential towards adipogenic differentiation. The morphological observation data was confirmed following gene expression analysis using RT-qPCR. The data revealed upregulations of FABP4 (more than 4 folds), PPARγ, and LEP (more than twofolds) for RP derived cells when normalized to those cells cultivated in BM. Similarly, more than twofold upregulated expressions for all adipogenic markers were found in the SC induced cells when normalized to those cells in BM. Interestingly, RP cells showed significant increases in FABP4 and PPARγ expression (*p* < 0.01) compared to SC derived cells. In contrast, SC derived cells showed a higher LEP expression (*p* < 0.05) compared to RP derived cells. On the other hand, LP induced cells displayed no expression for all adipogenic markers when normalized to those cells cultivated in BM (Fig. 4f–h).

**Fig. 5:**
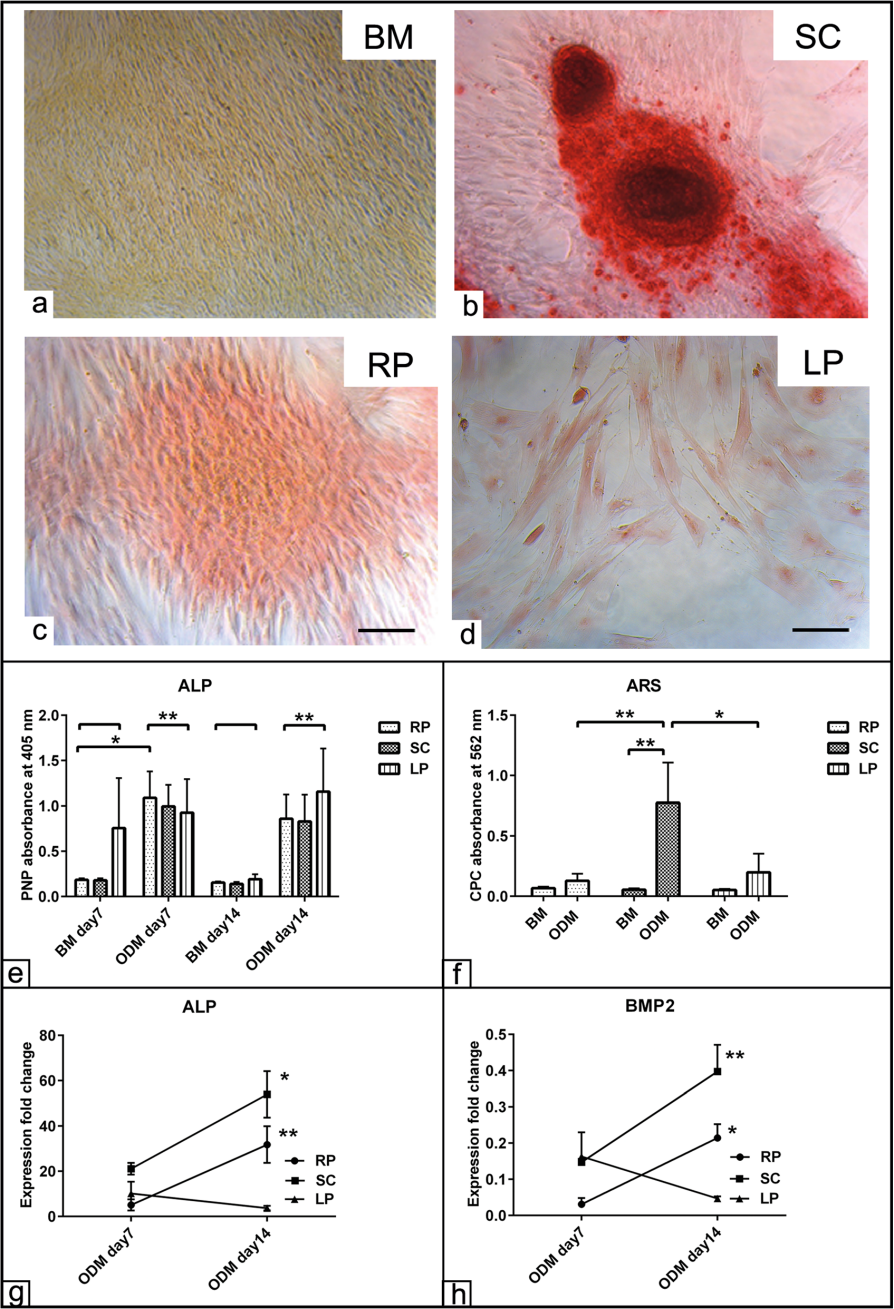
Detection of osteogenic differentiation of ASCs. ASCs harvested from subcutaneous (SC), retroperitoneal (RP), and lipoma (LP) fat sources were cultivated in the presence of osteogenic differentiation medium (ODM) for up to 21 days. **a**–**d** ASCs of SC, RP, and LP fat stained with Alizarin Red S (ARS) show osteogenic nodule formation and calcium deposition (red) in the mineralized matrix at day 21 post induction. Cells of the three groups were seeded in basal medium (BM). **e** Semi-quantification of alkaline phosphatase (ALP) activity as shown following osteogenic differentiation induction up to day 14 shows promoted ALP activity in all experimental groups including LP derived cells compared to those cells cultivated in BM condition. The p-nitrophenylphosphate is metabolized into p-nitrophenol (PNP) in the presence of ALP activity. The values of PNP induce a color change from colorless to yellow. **f** Semi-quantification of the bound Alizarin Red S (ARS) was measured by CPC at day 21 post osteogenic induction. Analysis of CPC absorbance at 562 nm revealed a higher osteogenic capacity of SC derived ASCs compared to RP and LP derived cells. **g**, **h** Expression fold change of ALP (**f**) and BMP2 (**g**) at days 7 and 14 post osteogenic differentiation induction shows upregulation of osteogenic markers in RP and SC derived cells compared to LP cells. The expression was normalized to non-induced cells in BM using 2^−∆∆cq^ method [30]. All data presented as mean ± SEM. *p < 0.05 and **p < 0.01. Scale bar = 100 μm

To evaluate the osteogenic differentiation potential of cells from all three fat sources, ALP activity indicative for osteogenic commitment as well as ARS staining for calcium deposition were assessed up to day 14 of differentiation. The morphological observation showed cellular clumping-like aggregations stained in red and was referred as osteogenic nodules in the presence of ODM as shown in RP, SC, and LP compared to those cells in the BM condition (Fig. 5a–d). A two-way ANOVA analysis demonstrated overall increases of the ALP activity in the cells from all fat sources at day 7 and day 14 (*p* < 0.01) post induction and compared to those cells cultivated in BM (Fig. 5e). Additionally, the bound ARS was dissolved using CPC assay. The quantification showed significant increases of ARS staining in the cells of SC fat (*p* < 0.01) compared to the relative BM condition. Similar observation in terms of higher ARS values was detected in the SC fat cells compared to RP (*p* < 0.01) and LP (*p* < 0.05) fat-derived cells. In contrast, the osteogenic induced cells of RP and LP revealed only slight but non-significant increases of ALP activity (Fig. 5f). Next, the relative osteogenic markers ALP and BMP2 expression was quantified using RT-qPCR. A two-way ANOVA revealed no expression was detected at day 7 post induction in all experimental groups. However, significant upregulation of ALP expression was found in RP (*p* < 0.01) and SC (*p* < 0.05) derived cells at day 14 compared to day 7 (Fig. 5g). Similarly, the expression of BMP2 showed no alteration at day 7 in all inductions; however, upregulated BMP2 expression was found in the SC (*p* < 0.01) and RP (*p* < 0.05) derived cells at day 14 compared to day 7 (Fig. 5h). The analysis demonstrated significant interaction (*p* < 0.05) between the effects of cell source in the course of osteogenic induction.

**Fig. 6:**
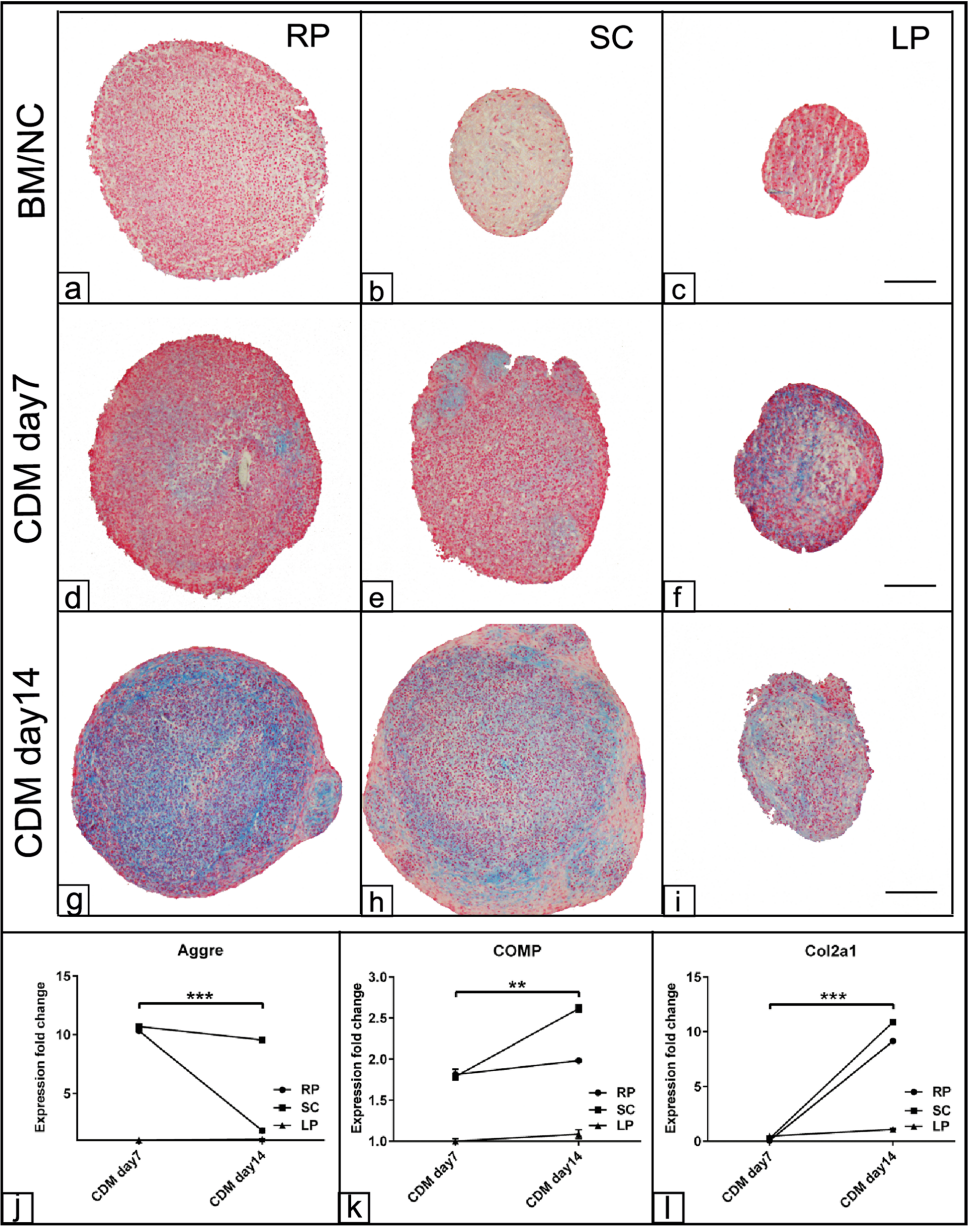
Evaluation of chondrogenic differentiation of ASCs. ASCs harvested from retroperitoneal (RP), subcutaneous (SC), and lipoma (LP) fat sources were cultivated as pellets in the presence of chondrogenic differentiation medium (CDM) for up to 21 day. The cell pellets were fixed in 4% PFA and were processed for histological examination. 5-μm sections from all experimental groups were stained using 1% Alcian blue. Photomicrographs of the cell pellet section at day 7 (**d**–**f**) and day 14 (**g**–**i**) show cartilage glycosaminoglycans (blue) indicative for chondrogenic differentiation. Sections of cells cultivated in basal medium were served as negative controls (BM/NC **a**–**c**). **j**–**l** Expression fold change of Aggre (**j**), COMP (**k**), and Col2a1 (**l**) at days 7 and 14 post chondrogenic differentiation induction show upregulation of chondrogenic markers in RP and SC derived cells compared to LP cells. The expression was normalized to non-induced cells in BM using 2^−∆∆cq^ method [30]. All data presented as mean ± SEM. **p < 0.01 and ***p < 0.001. Scale bar = 100 μm

In order to verify the chondrogenic potential of ASCs harvested from the three fat sources, the cells were differentiated into the chondrogenic lineage up to 14 days. As shown already after day 7 post induction, Alcian blue staining indicative for the cartilage glycosaminoglycans was observed in all experimental groups (Fig. 6d–f) compared to those cells cultivated in BM (Fig. 6a–c). At day 14, RP derived ASCs demonstrated more intensive blue staining compared to those cells harvested from either SC or LP fat (Fig. 6g–i). The morphological observation for the chondrogenic pellet of LP revealed smaller cell pellet with a weak Alcian blue staining compared to RP and SC cell pellets. Next, evaluation of the chondrogenic differentiation relative marker expression was carried out using RT-qPCR. The analysis showed up to 10-fold upregulation for Aggre expression in RP and SC derived cells at day 7 post chondrogenic induction when normalized to BM. The expression was sustained up to day 14 as shown in SC derived cells; however, it was downregulated for RP derived cells at day 14 (*p* < 0.001) compared to day 7 post induction (Fig. 6j). In contrast, a gradual upregulated COMP (*p* < 0.01) and Col2a1 (*p* < 0.001) expression was detected in RP and SC derived cells up to day 14 post chondrogenic induction compared to matched chondrogenic induced cells at day 7 (Fig. 6k, l). No expression was detected in LP derived cells following chondrogenic induction up to day 14.

## Discussion

Identification and selection of MSCs from an optimal tissue source are important issues in cell-based therapy. Although various sources are available for stem cell production, these do not necessarily lead to any significant difference in growth rate and differentiation potential at the desired target tissue after implantation. The present study aimed to characterize equine ASCs from different fat sources including lipomas to determine their potency for applications in equine regenerative medicine.

The macroscopic evaluation of the different adipose tissues revealed a marked difference with regard to tissue structure and tissue consistency between the subcutaneous and the retroperitoneal fat compared to abdominal pedunculated lipomas. The lipoma-derived tissue was dense compact mass with an outer fibrous capsule and inner adipose matrix. By contrast, the subcutaneous and retroperitoneal fat showed the typical morphological appearance of white adipose tissue with a softer consistence.

Apart from characterizing the gross morphological appearance, this study is among the first investigations giving a detailed analysis of equine stem cells isolated from different fat provenances as it has similarly been carried out for human adipose-derived cells [32] and for the whole stromal vascular fraction of various equine fat sources [33].

In the current study, adipose tissue was harvested from horses during emergency abdominal surgery. The standard median laparotomy approach gave access to sufficient or even abundant amounts of retroperitoneal fat which could be harvested without additional trauma to the patient and in sufficient amounts for several in vitro applications. Conclusively harvesting retroperitoneal fat, e.g., for future autologous or allogenous cell therapies, should be considered in horses that undergo elective or emergency laparotomy. Preferably, the fat should be harvested soon after the laparotomy to avoid trauma to the sample which is inevitable during abdominal exploration by the surgeon.

Mesenteric lipomas are often encountered during routine laparotomies. If they are an accidental finding, they usually have a pear-like shape and have a soft-elastic consistency. Intestinal strangulation is caused by pedunculated lipomas which may feel soft-elastic or firm. Lipomas harvested for the current study were pedunculated, and most of them had strangulated the small intestine and had a rather firm consistency. The firmness of the lipomas may correlate positively to their age and negatively to the potential of the derived ASCs.

A thorough characterization of cells of different fat sources is important as there is contradictory information about their quality and potency. It has been reported by some authors that there was no statistically significant evidence of fat source-related effects on the proliferation and differentiation potential of ASCs [34], while other investigators revealed that subcutaneous ASCs have greater differentiation potential compared with ASCs from other fat provenances suggesting the superiority of this fat source for regenerative approaches [35].

In compliance with the international society for cellular therapy (ISCT) [36], in the current study, ASCs from subcutaneous, retroperitoneal, and lipoma fat exhibited plastic adherence ability with a spindle-shaped fibroblast-like morphology as it has been similarly reported by [25, 26].

In addition to the morphological identification, MSCs must express the relative surface markers and show the tri-lineage differentiation potential [36]. Determination of stem cell marker expression such as CD44, CD105, and CD90 using PCR revealed a stronger expression of these transcripts in cells from retroperitoneal and subcutaneous derived ASCs than in LP cells. Furthermore, there were no detectable differences in the expression of these surface markers between ASCs from SC and RP fat. These results are partially contradictory to the findings reported in human ASCs. For example, CD44, a transmembrane glycoprotein, important for cell differentiation and involved in cell-cell and cell-matrix interactions, was expressed in human adipose tissue as well as in lipoma-derived cells [20]. In contrast, other study reported an even higher expression in lipoma tissue compared to normal adipose tissue [37].

Moreover, CD105, a membrane glycoprotein and part of the TGF-β complex, plays an important role for cartilage regeneration. The results showed reduced expression of CD105 in lipoma-derived ASCs compared to those cells of SC and LP fat. These findings are in line with similar observation in human lipomatosis [38]. However, in contrast, other reports demonstrated similar expression of CD105 in cells of various fat sources including lipoma [20, 32]. CD90 which is a common indicative surface marker especially to identify equine mesenchymal stem cells [29] showed lower expression in lipoma-derived cells compared to cells from the other sources. These data are in line with a study in canine ASCs comparing abdominal and subcutaneous fat sources which revealed that ASCs were positive for CD90 and CD44 [39]. As expected, the present study revealed a negative expression for CD45 and CD34 in cells from all fat sources as previously reported by the authors in equine [27] and by other groups in canine ASCs [39].

Investigation of cell viability and cell proliferation using the MTT assay revealed that cells from the retroperitoneal fat tissue have a higher proliferation capacity than those cells derived from subcutaneous fat tissue and lipomas. The increased cell viability as shown in RP derived cells was more likely due to the increases in cell number as indicated by the SRB assay. These data not only document the increased proliferation rate of RP derived ASCs but also show the phenotypic variability of ASCs from different fat sources. It has been reported that although there are no literature data available for lipoma-derived ASCs, it is known that variabilities in cell proliferation of different fat sources may exist [40]. In the same line, studies in the mouse and human have shown increased proliferation ability of visceral fat-derived ASCs when compared to cells derived from subcutaneous fat [15]. Thus, the proliferation capacity is an important aspect for the expansion of ASCs in order to achieve sufficient cell population readily for the use in regenerative therapies. Differences in cell proliferation have also been documented with respect to the tissue type cells were derived from: It has been found that ASCs show a higher proliferation rate compared to those cells from bone marrow and cartilage [41].

The CFU assay is used to define the self-renewing potency of a stem cell population and thus confirming stem cell characteristics, which gives a hint whether a cell population is suitable for a use in cell-based therapies. Thereby, it is a standard procedure to proof the occurrence of stem cells in the cell population [36]. In the present study, it was clearly shown that retroperitoneal and subcutaneous fat-derived cells have a similar CFU performance, while lipoma-derived cells showed an almost 50% reduction of CFU ability, indicating an impaired self-renewing potency. In a study comparing the effect of subcutaneous and visceral fat-derived ASCs on cardiac infarction, the authors revealed a higher proliferation rate and CFU ability of the subcutaneous compared to visceral fat-derived cells [42].

In many studies, it has been shown that stem cells from adipose tissue have a remarkable trilineage differentiation potency and are thus a good source for cell-based regenerative medicine [43]. Adipogenic differentiation of cells derived from the three fat sources was investigated using the Oil red O staining for the occurrence of lipid droplets. The quantification of the bound ORO staining unequivocally revealed that lipoma-derived ASCs have a significantly weaker capacity to differentiate into the adipogenic direction as confirmed by downregulated FABP4, PPARγ, and LEP expression compared to ASCs of RP and SC fat. Interestingly, cells derived from RP however showed a strong adipogenic differentiation potency compared to cells isolated from the subcutaneous fat source. A comparison between lipoma-derived ASCs and cells of other sources has also been made for human ASCs [32]. The authors reported an impaired adipogenic differentiation capacity of lipoma-derived cells compared to ASCs derived from the other fat tissue sources as indicated by adipocyte formation and the relative adipogenic marker expression suggesting a different mechanism regulating the differentiation of LP derived cells [32]. Such an observation was also reported in another human study revealing intrinsic variations between visceral and subcutaneous fat depots in both gene expression and differentiation potency [44]. Along the line, a study revealed even a regional and age-related variation in the lipolytic potency of the cells derived from different subcutaneous fat depots [34]. In contrast, it has been reported that the subcutaneous derived ASCs exhibited a higher adipogenic potential compared to those cells of visceral fat [15].

Despite these obvious differences between human and equine lipoma-derived ASCs, it has to be emphasized that for the current study, mainly firm pedunculated strangulating lipomas were selected. A comparison to ASCs from less mature mesenteric non-strangulating lipomas should be considered for future studies.

ASCs have proven a high potential for osteogenic differentiation which make them interesting candidates for bone tissue engineering [45]. Thus, osteogenic differentiation of ASCs from the three sources was investigated in our study by cultivating equine ASCs in an osteogenic induction medium for up to 21 days. Osteogenic differentiation was evaluated morphologically by tracking the osteogenic nodules as well as by semi-quantification of ALP activity and Alizarin Red staining for the detection of calcified inorganic matrix components. The present results revealed increased ALP activity for up to day 14 in all experimental groups compared to BM. However, matrix mineralization indicative for calcium deposition was only detected in cells derived from the subcutaneous fat tissue. These data were in parallel with upregulated ALP and BMP2 expression. Similar to our findings, a canine study comparing the differentiation capacity of ASCs from subcutaneous and visceral fat revealed a higher matrix mineralization with subcutaneous fat-derived cells compared to those cells from visceral fat [39]. In the same line, it has been reported that both lipoma- and adipose-derived cells exhibited different stages of osteogenic differentiation after 16 days post induction suggesting the molecular background of both cell types might control their differentiation fate [32]. A recent report documented similar results in terms of increased ALP activity indicative for osteogenic commitment in the lipoma-induced cells with no alteration in the ARS staining compared to fat-derived ASCs [46].

Interestingly, in the current study, the expression of osteopontin (OPN) using RT-qPCR was only detected in the lipoma non-induced ASCs compared to both subcutaneous and retroperitoneal fat-derived cells. Generally, OPN plays an important role in the effect of unloading-induced alterations on the differentiation of bone marrow into osteoblasts and osteoclasts [47]. Additionally, OPN promotes osteoblast adhesion, differentiation, and function and is therefore important for bone metabolism [48, 49]. The expression of OPN in lipoma cells suggests the tumor phenotype of these cells compared to those cells derived from fat. It has been reported that OPN RNA and protein were strongly expressed in the cells of lung tumors compared to normal lung tissue [50]. These data were confirmed when we examined CA9 expression. The analysis revealed a strong expression of the tumor marker CA9 which was detected only in LP derived cells (*p* < 0.05) compared to those cells from RP and SC fat. These results suggest that despite of the tumor phenotype of the lipoma-derived cells, there is a capacity for the differentiation into the osteogenic lineage by lipoma-derived cells. In fact, OPN is a late osteogenic marker produced in bone by osteoblasts during premineralization [51]. This might also be the explanation for its expression exclusively in lipoma-derived cells, as there are numerous studies on lipoma tissue ossification observed in various organs [52–54] and was frequently addressed as osteolipoma [55].

The evaluation of the chondrogenic potency of ASCs revealed a higher capacity of RP and SC fat-derived cells towards cartilage formation as shown by Alcian blue staining and upregulation of Aggre, COMP, and Col2a1 expression up to day 14 compared to LP derived cells. Similar study has shown that mechanical stress via centrifugation enhanced chondrogenic differentiation as indicated by the upregulation of Aggre, Col2a1, and collagen type I [56]. In agreement with our data, a study has documented the chondrogenic potential of ASCs from SC fat [57]. Thus, the data from us and others suggest that ASCs from SC or RP fat could be a potential therapeutic candidate for cartilage repair and tissue engineering in equine.

## Conclusion

Our study aimed to investigate and compare stem cell quality of cells derived from different adipose tissue sources including lipoma in order to get further insight into stem cell biology and to obtain information, which stem cell population is preferential for a therapeutic application in equine regenerative medicine. In this respect, although RP fat-derived cells demonstrated higher proliferation and adipogenic differentiation capacities, SC derived cells showed a higher osteogenic potential. Additionally, both RP and SC fat-derived cells were able to induce chondrogenic differentiation compared to those cells of LP. The initial expectation was that lipoma-derived cells may have a higher proliferation rate and also a better differentiation potential compared to cells from the other two fat sources. However, the present study has clearly shown that equine lipoma-derived cells are not suitable for tissue engineering approaches. The data suggest that the molecular regulation for lipoma-derived cells is completely different compared to other fat sources. Moreover, we have spotted a difference in the differentiation pattern between ASCs from SC and RP normal fat source that would require further investigation.

## Data Availability

The data collected and the analysis performed to generate the manuscript results are available from the corresponding author on reasonable request.
